# Absence of the Lumbosacral Trunk

**DOI:** 10.7759/cureus.1809

**Published:** 2017-10-30

**Authors:** Cameron K Schmidt, Joe Iwanaga, Emre Yilmaz, Charlotte Wilson, Rod J Oskouian, R. Shane Tubbs

**Affiliations:** 1 Clinical Anatomy, Seattle Science Foundation; 2 Seattle Science Foundation; 3 Swedish Medical Center, Swedish Neuroscience Institute; 4 Neurosurgery, Complex Spine, Swedish Neuroscience Institute; 5 Neurosurgery, Seattle Science Foundation

**Keywords:** lumbosacral trunk, lumbosacral plexus, anatomic variation, cadaver

## Abstract

The lumbosacral trunk, typically comprised of part of the fourth lumbar ventral rami and the entirety of the fifth lumbar ventral rami, serves as a connection between the lumbar and sacral plexuses. Developmental differences underlie the variable relative contributions of L4 and L5 to the lumbosacral trunk. Herein, we report a rare case in which dissection of an adult male cadaver revealed no L4 contribution to the lumbosacral plexus. We discuss the surgical and clinical implications of such an anatomic variation.

## Introduction

The lumbosacral trunk is typically formed by the ventral rami of part of the fourth and the entirety of the fifth lumbar spinal nerves [1]. Traveling medial to the psoas major, the lumbosacral trunk descends against the ala of the sacrum, crosses the pelvic brim medial to the sacroiliac joint, and joins the S1 nerve root, thus uniting the lumbar and sacral plexuses i.e., lumbosacral plexus [1-3]. Anatomical variations in this region result in variable relative contributions of L4 and L5 to the lumbosacral trunk.

We herein present a cadaveric case report in which the L4 nerve did not converge with L5 to form the lumbosacral trunk, resulting in no L4 contribution to the lumbosacral plexus.

## Case presentation

**Figure 1:**
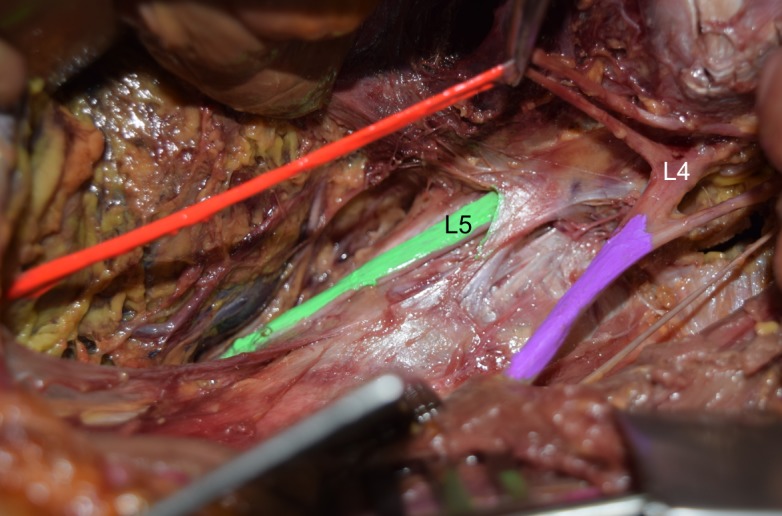
Cadaveric dissection of the left lumbar plexus. Note the absence of L4 ventral ramus (L4) fusing with the L5 ventral ramus (L5 and colored green) i.e., absent lumbosacral trunk. For reference, note the obturator (orange) and femoral nerves (purple).

We report on a 79-year-old male cadaver who died of a myocardial infarction. There was no history of abdominopelvic surgery or disease. No surgical incisions were found on the back or abdominal wall. During routine dissection of the left posterior abdominal wall following piecemeal removal of the psoas major muscle, it was noted that the lumbosacral trunk was absent. In other words, the L4 ventral ramus was found to not communicate with the L5 ventral ramus (Figure 1).

The last major fibers from L4 were found to enter the obturator and femoral nerves. In this specimen, L5 continued into the pelvis to mix with the S1 ventral ramus and was the only lumbar nerve to contribute to the lumbosacral plexus. No other neural (e.g., conjoined, duplicated nerve roots) or vascular variations were noted in this specimen. No transitional vertebrae were identified, and the right lumbosacral trunk was present.

## Discussion

The lumbar plexus lies anterior to the transverse processes of L1 through L3. Caudal to the lumbar plexus, the sacral plexus lies against the anterior aspect of the piriformis, external to the pelvic fascia. The sacral plexus is composed of the lumbosacral trunk, the ventral rami of S1 through S3, and part of the ventral ramus of S4 [1]. The lumbosacral trunk, formed by the union of the ventral rami of part of L4 and all of L5, serves as a bridge between the lumbar and sacral plexuses [4]. Emerging on the medial aspect of the psoas major, the lumbosacral trunk courses over the pelvic brim to merge with the ventral ramus of S1, thereby providing the lumbar contribution to the lumbosacral plexus [2].

The length of the lumbosacral trunk ranges from 21.0 to 39.0 mm in males and 26.0 to 38.0 mm in females [2]. In a 31-cadaver study conducted by Waikakul and colleagues (2010), the union of L4 and L5 was found to occur at (25.8%), above (35.5%), or below (38.7%) the most anterior part of the sacroiliac joint [3]. In up to 16% (8/50) of cases, L4 merges with L5 below the pelvic brim [5]. Atlihan, et al. (2000) dissected 60 cadaveric specimens and reported an average distance of 11.49 ± 2.71 mm between the superior aspect of the sacroiliac joint and the beginning of the lumbosacral trunk. The same study found the lumbosacral trunk to be an average of 5.31 ± 2.16 mm from the sacroiliac joint at the pelvic brim and 12.57 ± 3.45 mm at the sacral promontory [6]. These values are in accord with those reported by Ebraheim and colleagues [7].

The relative contribution of L4 to the lumbosacral trunk displays wide variation. Webber describes cases ranging from minimal L4 contribution to those in which the entire L4 ventral ramus converges with L5 [5]. While L5 is thicker than the contributing branch of L4 in most cases, the branch from L4 to L5 has been shown to exceed the thickness of L5 itself in 21.6% (11/51) of cases [4]. Anatomical variations in which L3 is an additional contributor to the lumbosacral trunk have also been reported [4-5].

Due to this variability, L4 often serves as a boundary root, demonstrating greater participation in the lumbar or sacral plexus. Depending on proportionate participation, the lumbosacral plexus can be classified as cranially prefixed or caudally postfixed. A sufficiently postfixed plexus may explain the lack of L4 merger with L5, although this was not found in our case. In his study of 50 cadavers, Matejčík (2010) reported a 9.8% (5/51) rate of postfixed plexuses, yet the L4 root was present in the lumbosacral trunk in all 51 cases [4].

In a study of 60 specimens, Atlihan and colleagues (2000) found four cases (6.66%) in which the L4 nerve failed to merge with L5 [6]. Similarly, Webber (1961) reported that 6% (3/50) of cases demonstrated no L4 contribution to the lumbosacral trunk [5]. These variations are the likely product of aberrant development.

Importantly, the lack of L4 merger with L5, and the corresponding lack of L4 contribution to the lumbosacral plexus in the absence of a postfixed plexus holds clinical and surgical implications. In such cases, clinical examination of L4 function within the sacral plexus would yield results suggestive of an L4 injury. Similarly, knowledge of this anatomical variant is important for the surgeon in interpreting neuromonitoring during lateral interbody fusion surgery that involves retraction or entering the psoas and in determining the origin of any post-operative deficits.

## Conclusions

The lumbosacral trunk is a structure typically composed of a part of the ventral ramus of the L4 nerve and the entire ventral ramus of the L5 nerve. In this case, dissection demonstrated the L4 nerve failed to merge with L5, thus depriving the lumbosacral plexus of its L4 contribution. Despite a low reported incidence in the literature (approximately 6%), such an anatomic variation holds clinically and surgically salient implications.
